# A Subtype Perspective on Cognitive Trajectories in Healthy Aging

**DOI:** 10.3390/brainsci14040351

**Published:** 2024-04-01

**Authors:** Emma A. Rodrigues, Gregory J. Christie, Theodore Cosco, Faranak Farzan, Andrew Sixsmith, Sylvain Moreno

**Affiliations:** 1 School of Interactive Arts and Technology, Simon Fraser University, Surrey, BC V3T 0A3, Canada; 2 Circle Innovation, Simon Fraser University, Surrey, BC V3T 0A3, Canada; 3 Department of Gerontology, Simon Fraser University, Vancouver, BC V6B 5K3, Canada; 4 School of Mechatronics and Systems Engineering, Simon Fraser University, Surrey, BC V3T 0A3, Canada

**Keywords:** trajectories, cognitive health, healthy aging, subtyping, heterogeneity

## Abstract

Cognitive aging is a complex and dynamic process characterized by changes due to genetics and environmental factors, including lifestyle choices and environmental exposure, which contribute to the heterogeneity observed in cognitive outcomes. This heterogeneity is particularly pronounced among older adults, with some individuals maintaining stable cognitive function while others experience complex, non-linear changes, making it difficult to identify meaningful decline accurately. Current research methods range from population-level modeling to individual-specific assessments. In this work, we review these methodologies and propose that population subtyping should be considered as a viable alternative. This approach relies on early individual-specific detection methods that can lead to an improved understanding of changes in individual cognitive trajectories. The improved understanding of cognitive trajectories through population subtyping can lead to the identification of meaningful changes and the determination of timely, effective interventions. This approach can aid in informing policy decisions and in developing targeted interventions that promote cognitive health, ultimately contributing to a more personalized understanding of the aging process within society and reducing the burden on healthcare systems.

## 1. Introduction

Cognitive aging refers to the dynamic and variable longitudinal changes in cognitive function that occur throughout the aging process [1]. While extensive research has focused on identifying severe cognitive loss, less is known about the trajectories associated with healthy cognitive aging [2]. Within this field, most studies have focused on characterizing normative cognitive aging trajectories. For instance, the work of Salthouse (2019) investigated the trajectory of normal cognitive aging by comparing age trends with quasi-longitudinal, cross-sectional, and longitudinal methods [3]. The author highlighted the need to better model the healthy aging population beyond the view of pathology and argued methodological strategies to achieve this goal. Similarly, Tucker-Drob (2019) and Wilson et al., (2020) discussed how to best characterize the trajectories of normative cognitive aging and highlighted the need to distinguish the contributions of pathological processes from those of normative degenerative processes [4,5]. Despite the methodological advances and the valuable distinction between the healthy and pathological cognitive trajectories described by Salthouse (2019), Tucker-Drob (2019), and Wilson et al., (2020), this paper critiques the limitations of current normative models and discusses alternative approaches that can capture individual-specific dynamics in cognitive trajectories in healthy aging [3,4,5].

Although the proposed approaches model the population as a whole, important idiosyncrasies relevant to alternative trajectories may not be captured. Authors, such as Molenaar (2015), Moreno et al., (2023), and Boker, Molenaar and Nesselraode (2009), demonstrated that the factors identified to characterize a group do not necessarily apply to each individual within that group [6,7,8]. Consequently, this implies that the inter-individual variations considered in the model, which account for differences between individuals, do not explain the intra-individual changes [8]. Inter-individual variability refers to the possible cognitive differences between two or more individuals, whereas intra-individual variability reflects the individual changes that occur over time. In other words, the factors that explain the variability between individuals in the group are not sufficient to explain the changes that occur within an individual over time. In fact, when considering individual dynamics beyond the normative pattern, important information may be lost [9]. This is particularly important when studying the older adult population given the substantial heterogeneity in late-life healthy cognitive trajectories, as some individuals experience little change while others experience complex non-linear dynamics, making it difficult to model change uniformly [2,10]. This results in the proposed modeling approaches of normative healthy cognitive aging not being inclusive of healthy individuals whose cognitive trajectories may display different dynamic behaviors.

Mella et al., (2018) provided a possible solution through the characterization of a myriad of individual trajectories of cognitive health. However, their approach led to challenges in obtaining a reliable assessment of intra-individual variability [9]. A lack of reliability results from increased noise obtained when collecting information over time under different conditions (e.g., time of day of assessment, caffeine intake, nicotine intake, sleep deprivation, and nervousness).

## 2. Subtyping in Healthy Cognitive Aging

Given the limitation of normative modeling and the proneness to the error in assessing individual change, we propose an analysis of disaggregated cognitive profiles of the population in more homogenous subtypes [7]. We propose a subtyping approach with the goal of identifying commonalities within the population that still capture key individual features while also reducing the noise of the sample. In a literature review, Wu et al., (2020) supported this approach, having explored multiple subtypes of cognitive trajectories [2]. The authors highlighted the detection of trajectories that included (i) relative stability, (ii) high-functioning individuals, and (iii) declines to varying extents. This review examined work that identified different numbers of cognitive trajectory subtypes. The authors identified three to six subtypes within the healthy older adult population. The presented subtypes are described in terms of their stability and decline rates. The authors that identified three subtypes described them as “stable”, “slowly declining”, and “rapidly declining” (e.g., [11,12,13]). The authors that identified a different number of trajectories had similar descriptions up until six trajectories, which were described as “highest with minor increase”, “high with minor increase”, “medium with minor increase”, “medium stable”, “low with major decline”, and “lowest with major decline” (e.g., [14,15]). The differences in the samples used, methodological approaches, and selection of cognitive assessments may justify the variations in the number of subtypes presented by the different studies.

Overall, there is ongoing debate in the literature regarding the most appropriate approaches for studying cognitive trajectories in aging. It has been widely acknowledged that linear models may not fully capture the complex changes observed in cognitive aging [16,17,18]. Moreover, the notion of a ‘one-size-fits-all’ approach has been questioned, as it may fail to account for the significant heterogeneity present within the healthy older adult population [12,19,20,21]. However, a disconnect exists between the conceptualization of the cognitive aging process and the methodological approaches employed, resulting in a lack of consensus across different fields and studies [22,23,24]. In our current work, we emphasize that cognitive aging is a dynamic, non-linear process characterized, in part, by its inter- and intra-individual heterogeneity [23]. While some individuals may exhibit meaningful declines that are not captured using pathology screeners, others may reach peaks in cognitive performance later in life [5,25]. Despite these differences, an approach that considers population subtyping would allow us to capture cognitive changes at differentiated levels. Furthermore, it would reduce the noise associated with individual tracking while still integrating important differences associated with the healthy aging population. Lastly, subtyping exposes windows of opportunity that allow the reversal of smaller declines in cognitive health, preventing greater ones [3]. The reason for this is that subtyping allows for the identification of different trajectories to be considered within the healthy cognitive aging population rather than using a single model. This preliminary evidence suggests that subtyping can reveal distinct cognitive trajectories that are not captured using normative modeling approaches as it rejects the one-size-fits-all approach, and as such, changes in individual trajectories are easier to identify within each population subset.

## 3. Cognitive Trajectories in Healthy Aging

In order to identify the different subtypes present in the population trajectories, it is necessary to understand what motivates change and how this can be captured. Blazer, Yaffe, and Karlawish (2015) described that the age-dependent changes in cognitive function are due to longitudinal differences in experiences, health status, lifestyles, education, attitudinal and emotional factors, socioeconomic status, and genetics [26]. Further, the contributions of each of these factors are heterogeneous across the population, making it challenging to quantify their exact contributions [27]. Research indicates that the heritability of cognitive health shifts over the lifespan [28]. Twin studies, such as Finkel and Reynolds (2009) and Plomin and Deary (2015), suggested that heritability increases from childhood to adulthood (contributions ranging between 40% and 80%) but decreases in old age (contributions ranging between 40% and 60%) [29,30]. Other studies have proposed heritability estimates ranging from 31% to 51% in older adults [31]. This decline in heritability with age may be linked to heightened responsiveness to environmental influences [32,33]. Research by Deary et al., (2012) indicated that approximately 75% of cognitive ability variance from childhood to old age is attributed to environmental factors [34]. While the exact contributions to cognitive performance remain uncertain and vary among individuals and over time, it is widely acknowledged that these factors play a significant role in shaping cognitive trajectories in older adults [35,36]. The way in which these changes are typically detected is through pathology screeners (such as the mini-mental state examination (MMSE)). Thus, when severe, these changes can be considered as symptoms of pathological conditions. If the changes are small, relative to the differences associated with the pathology, they are more likely to go unnoticed. However, gradual changes that accumulate over time could have detrimental effects on quality of life [3].

**Figure 1:**
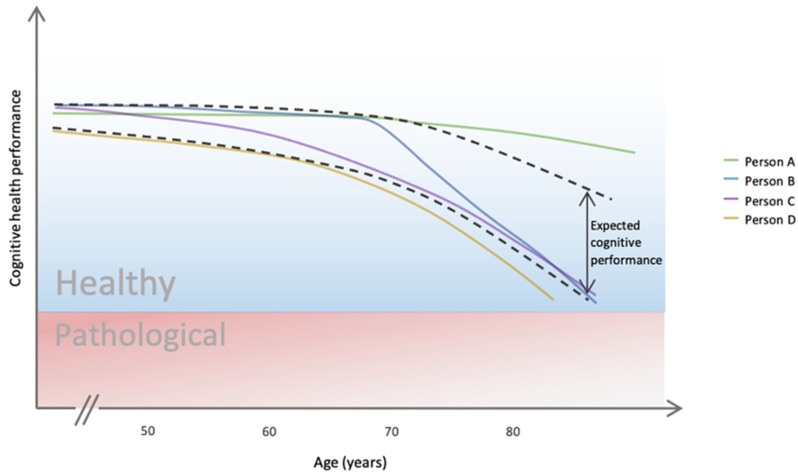
Examples of cognitive trajectories in healthy aging.

Figure 1 depicts a representation of individuals from different possible subtypes present in the healthy aging population. This representation draws from studies by Kim et al., (2023) and Wu et al., (2020), emphasizing the value of subtyping for understanding cognitive trajectories in aging [2,37]. Kim et al., (2023) advocated for subtyping to identify homogeneous subpopulations within a heterogeneous group. Similarly, Wu et al., (2020) highlighted the importance of classifying participants with similar cognitive trajectories to address population heterogeneity. Despite emerging studies, limitations persist, such as inadequate sample compositions [38] and sub-optimal cognition measures [12,39]. Notably, comparing younger and older adults may neglect the meaningful differences among older individuals, and using screening tests such as the MMSE may not capture healthy cognitive performance effectively [40]. The choice of three to four trajectory clusters with distinct profiles used in Figure 1 is aligned with what is typically identified in studies (e.g., [12,39,41,42]). The dashed lines exemplify the range of expected cognitive performance within which Salthouse’s (2019) normative trajectory would fall [3]. Here, considering multiple subtypes that include alternative rates and times of change can lead to a better capture of significant deviations from a healthy trajectory. Figure 1 also illustrates how certain scoring thresholds (often used in pathology screeners) can lead to misleading, binary views on healthy cognitive aging. Figure 1 illustrates that within healthy cognitive aging, a range of distinct possible trajectories can be considered. Given the complexity and multidimensional nature of the cognitive aging process, a categorization of “healthy” or “non-healthy” based on a single cutoff score may be overly simplistic [19,23]. The variability present among cognitively healthy individuals manifests in factors such as current performance level [43], magnitude of age-related changes [44], rate of decline [19], and susceptibility to future changes [45]. Scoring thresholds fail to capture this heterogeneity, as they consider cognitive aging as binary. Instead, Figure 1 illustrates the need for a more nuanced, multifaceted approach that captures the variability and individual differences within the healthy aging process. To that end, a more comprehensive, longitudinal assessment is needed to fully understand an individual’s cognitive aging trajectory and identify appropriate interventions to minimize declines and support cognitive health [19].

Let us consider people A, B, C, and D—green, blue, purple, and yellow, respectively. These lines represent four different illustrative individuals with distinct cognitive trajectories. These individuals reflect possible subtypes of the population. From Figure 1, the three individuals—people A, B, and C are considered cognitively healthy. The fourth individual, person D, is also considered cognitively healthy but is outside of the expected range.

Without a subtyping approach (contrasted with current normalized or individualized approaches), it becomes unclear whether the observed individual-specific dynamics are meaningful, as the current measures may not be sufficiently granular. For example, the trajectory of person A is potentially not captured accurately due to ceiling effects. Additionally, person B appears to be a high-performing adult, though they suffer from a sudden change in the decline rate, which, depending on the timing of the measurement, can go unobserved until there is a drastic behavioral manifestation of this decline. This is of particular importance given the individual non-linear changes in cognitive trajectory that may take place over time [46]. Lastly, C and D appear to have a relatively constant declining rate with increasing age. However, person D has a cognitive trajectory that is always close to the lower threshold, in which normal aging is expected to occur, but is also still far from the functioning threshold. Despite these different cognitive trajectories (notably, the different decline rates), all these individuals would be considered in good health. Thus, no recommendation would be given to mitigate or reverse their individual trends. Unless an individual drops below a threshold defined a priori, their degree of cognitive health is not typically explored further. Contrary to pathological cognitive aging, it is necessary that healthy cognitive aging is monitored over time, as one measurement is not sufficient to understand the rate of individual decline (e.g., a score below 23 on an MMSE assessment would warrant a diagnosis of dementia).

**Figure 2:**
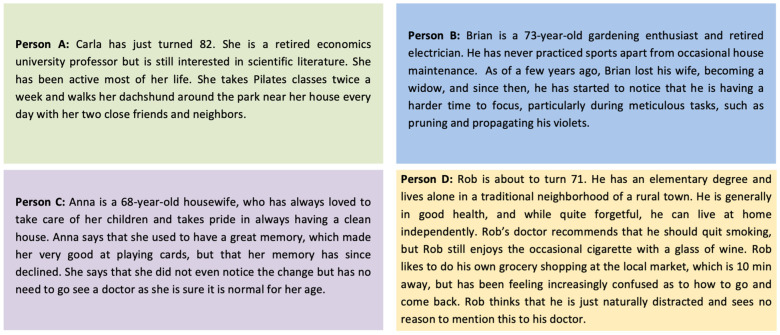
Personas associated with each presented cognitive trajectory. Person A, described in the green box, refers to person A in Figure 1, characterized by the green trajectory of sustained high cognitive performance. Person B, described in the blue box, refers to person B in Figure 1, characterized by the blue trajectory of sudden decline at the age of 70. Person C, described in the purple box, refers to person C in Figure 1, characterized by the purple trajectory of relative decline within the expected range of cognitive performance. Person D, described in the yellow box, refers to person D in Figure 1, characterized by the yellow trajectory of relative decline below the expected range of cognitive performance.

Example-based descriptions were created using personas to better illustrate how the different trajectories may impact the lives of healthy older adults. This allowed us to understand, through characteristics and difficulties, how the changes in cognitive trajectories may impact older adults rather than only exploring theoretical changes in cognitive health. Each persona described in Figure 2 is linked to the trajectories presented in Figure 1. These personas can be observed in Figure 2.

Overall, Figure 1 highlights (and Figure 2 exemplifies) how the early detection of steep cognitive changes, even in high-functioning adults, may be indicative of a fast decline into pathology (person B) or how slow declines nearing the lower scores of normal aging (person D) may require alternative recommendations. To prevent or delay disorders, such as dementia or other pathologies, individuals should not have to rely on pathology-specific screening tools, nor should they have to wait until symptoms present themselves. This subtype approach would allow for a faster and more efficient distancing from the likelihood of a diagnosis.

The notion of subtypes has been extensively described in the pathological aging literature to describe differences across individuals in the preclinical stages of dementia, within mild cognitive impairments (MCIs) and within dementia diagnoses. Sperling et al., (2011) described a range of different biomarkers that may be indicative of pathological cognitive decline [47]. Within the preclinical stage of dementia that precedes MCI, not all individuals with evidence of Alzheimer’s disease (AD) within the pathophysiological process will progress to clinical AD, highlighting the differences in responses to the biomarkers individuals may present with [47]. Stephan et al., (2012) discussed the vast variability within the MCI population and highlighted subtypes, such as amnestic MCI, non-amnestic MCI, and multi-domain MCI [48]. The authors highlighted that MCI carries vast heterogeneity and that each neuropsychological subtype previously introduced may reflect a different neuropathological profile [48]. Dementia is an umbrella term that can refer to a range of distinct disorders, of which the most common is AD. Less common dementing subtypes include Lewy body disease, vascular dementia, and frontotemporal dementia [49]. Within each of these examples, depending on the subtype that individuals belong to, different recommendations and treatments will be provided. This work furthers this conceptualization through the inclusion of a temporal dimension. Given the progressive properties of preclinical neuropathology, it may be of value to explore how subtyping trajectories can be considered within this framework. The applicability of the subtyping trajectory approach within the different stages of the pathological cognitive process is a topic of valuable consideration that is beyond the scope of this commentary. Given its relevance, a comprehensive review paper should be dedicated to this topic.

## 4. Reconciling Conceptual Understanding and Methodological Implementation

The current paper highlights the need for a better understanding of the cognitive changes associated with healthy aging. Salthouse (2019) provided a critical analysis that examined the overall trajectory of cognitive aging [3]. The author compared age trends in three distinct types of data: cross-sectional, longitudinal, and quasi-longitudinal. The motivation for using these types of data lies in the fact that both cross-sectional and longitudinal data are abundantly discussed in the literature but have key limitations. Quasi-longitudinal data is here discussed as a potentially viable alternative that involves the comparison of individuals from the same birth cohort, though evaluated at different times. Salthouse highlighted the need to better model the healthy aging population beyond the view of pathology and provided robust methodological strategies to achieve this goal. In this work, the authors propose a normative approach to studying cognitive aging, which may overlook smaller individual changes. We build on Salthouse’s (2019) work by discussing the relevance of capturing cognitive changes at the individual level and identifying key timings for appropriate recommendations [3].

Here, we highlight that the approaches used to study healthy cognitive aging cannot be the same as those used to study pathological aging. When describing pathological cognitive aging, absolute differences are typically sufficient to identify whether an individual is or is not diagnostically ill. However, the same approach is not suitable when investigating healthy cognitive aging. The reasons for this are threefold:

1. First, absolute scores are insufficient to determine the degree of cognitive health. Cognitively healthy individuals with similar scores on a pathology screener may not be significantly different, whether behaviorally or functionally. The reason behind setting these thresholds is to ensure an accurate diagnosis of the pathology while also ensuring the specificity and sensitivity of the tool to the pathology it was designed to detect. However, this further motivates why these may be inaccurate when identifying degrees of cognitive health in healthy older adults;
2. Second, even when using a cognitive test (not specific to a pathology), comparisons to normative trajectories may not be sufficiently informative of individuals’ health. The cognitive trajectory of an individual is dependent on the interaction between the environment, genetics, and individual choices, which may result in a distinct trajectory from the norm. Given the vast heterogeneity of the population, it is crucial to understand how much of the differences observed across individuals are due to inter-/intra-variability or whether the individuals are sufficiently distinct that they warrant different classifications, justifying the use of classification-specific solutions. This leads to the need to subtype the population so that population variability and individual dynamics may be better understood. As examples, in addition to Wu et al., (2020) and Kim et al., (2023) previously described, Rodrigues et al., (2022) subtyped the population depending on whether older adults were environment-resistant or environment-sensitive [2,37,45]. Moreover, Huo et al., (2020) studied the metabolic subtypes of cognition in aging [50]. Eavani et al., (2018) and Nyberg et al., (2023) subtyped individuals according to brain structure and function [51,52]. Finally, Lowe et al., (2019) identified subtypes in the older adult population in regards to brain connectivity [53]. These examples highlight the benefits of considering a subtyping approach, given the heterogeneity inherent to this population. Here, we propose a subtyping approach that includes a temporal dimension, allowing for the distinction of clusters of individuals based on cognitive scores as well as rates of change within each cognitive trajectory subtype. In the context of cognitive aging and given the need for the early identification of meaningful changes, subtyping applied to cognitive trajectories can help identify, within each subgroup, whether the observed changes are expected or unexpected and intervene accordingly. This approach is introduced with a focus on the study of cognitive trajectories, classifying individuals into different groups based on the similarities of how individual cognitive scores change over time [37]. Subtyping cognitive trajectories within healthy cognitive aging allows for an improved understanding of the expected cognitive trajectories, as it recognizes that different cognitively healthy individuals may have distinct patterns of cognitive change over time [39]. Overall, the proposed subtyping approach enables the identification of subgroups of individuals who share similar cognitive scores as well as similar patterns of change within their cognitive performance. This approach goes beyond analyzing the average cognitive performance across a population and provides a framework for capturing individual differences and temporal dynamics within cognitive trajectories;
3. Lastly, repeated assessments of cognitive function are more likely to identify important individual changes over time and their drivers [54,55]. Exploring individual rates of change can lead to identifying the impact of specific events (e.g., menopause) on cognitive trajectory and mitigate/minimize declines linked to the healthy aging process. Further, as there is a constant interaction between genes and the environment, repeated assessments would allow for a better understanding of the iterative feedback that may contribute to progressively shaping an individual’s cognitive trajectory. A final consideration is that the authors suggest a minimum of three time points to determine non-linear cognitive trajectories (e.g., [2]). However, it is important to ensure that practice effects are avoided. The magnitude of practice effects depends on the characteristics of the test itself as well as the test-taker [56]. These authors describe that individuals with fewer years of education may have an increased magnitude of practice effects due to repeated cognitive assessments.

The novelty of this paper lies in the proposal of a subtyping approach to cognitive trajectories in healthy aging, aiming to bridge the gap between conceptual understanding and methodological implementation. Authors such as Rodrigues et al., (2023) emphasized that cognitive aging is an individualized and variable process that changes over time [23]. Lindenberger (2014) discussed the contribution of meaningful environmental opportunities and constraints to this variability [57]. Further, Wu et al., (2020) highlighted the importance of considering population heterogeneity and temporal changes in identifying cognitive trajectories [2]. Despite these conceptual agreements that highlight the fundamental differences within the healthy older adult population, there exists a disconnect between this understanding of the cognitive aging process and the methods employed. Several authors have described healthy cognitive aging in the context of pathology, suggesting deviations from a normative trajectory as potential indicators of pathology (e.g., [3,4,58,59]). As a result, all healthy older adults are deemed the same and, as such, assessed as a whole. Conversely, some authors, such as Mella et al., (2018) and Fletcher et al., (2018), focused on individual cognitive trajectories [9,60]. As a result, all healthy older adults were deemed different and, therefore, assessed individually. This approach carries key limitations, including the increased noise associated with such assessments, as highlighted by [61]. To address this gap, the proposed subtyping approach seeks to reconcile the conceptual understanding of cognitive aging with practical methodologies. This approach identifies commonalities within the population while considering the heterogeneous process of cognitive aging, leading to a better understanding of the expected cognitive trajectories and early identification of meaningful changes.

Ultimately, the identification of subtypes, either through the use of cognitive trajectories or through alternative strategies, requires robust methodologies that can handle the complexity associated with the older adult population. This approach may lead to different recommendations depending on the changes observed. It is important to highlight that these identifications and recommendations are only possible with the close monitoring of cognitive health, one that will allow for the capture of possible individual change. Krakovska et al., (2021) discussed the value of considering lifestyle changes as possible recommendations given that “as individuals age, their cognitive health is less influenced by historical factors and is increasingly influenced by more immediate factors such as daily tasks and leisure activities” [32]. Following these findings, two examples of possible recommendations are listed below:

1. In the illustrative example provided in Figure 1, person B appeared to present a fast decline, and as such, recommendations should include participation in social prescribing programs in an attempt to minimize the slope of the decline rate. Alternatively, person A appeared to present a stable high trajectory, so no recommendations may need to be provided.
2. When describing the environment-sensitive and environment-resistant population, a possible recommendation could be that environment-sensitive individuals (those extremely sensitive to their surrounding environment) should be cautious and avoid negative factors such as sedentarism and smoking, as the consequences of these factors will be amplified when compared to environment-resistant peers (those extremely resistant to their surrounding environment). Similarly, they should search for positive factors such as concentration-driven tasks (e.g., reading and word games), as the benefits of these will also be amplified when compared to their environment-resistant peers [45].

## 5. Methodological Considerations for Subtyping Trajectories

Examples of methodological approaches used include cluster analysis (e.g., [62,63]), latent class analysis (e.g., [64,65,66]), and trajectory analysis (e.g., [39,42,67]), which have been used to identify subtypes within a population. These methods aim to identify groups of similar individuals based on their characteristics or patterns of response. However, the choice of method should be driven by several key considerations. Firstly, sample size plays a crucial role, as small sample sizes may hinder the detection of meaningful subtypes (resources such as UK Biobank (https://www.ukbiobank.ac.uk/ (accessed on 30 March 2024)), the health and retirement study (https://hrs.isr.umich.edu/about (accessed on 30 March 2024)), and the English longitudinal study of aging (https://www.elsa-project.ac.uk/ (accessed on 30 March 2024)), help to ensure more efficient and less bias estimates). Further, depending on the available data, the presence of individual contextual factors may result in the identification of specific subtypes that would not be observed among individuals with distinct contextual factors. Secondly, sample composition should be considered, as broader or heterogeneous samples, may lead to larger differences being detected, such as young vs. old or healthy vs. unhealthy, rather than capturing smaller, meaningful differences within specific subgroups. Thirdly, data quality is important, as missing data, measurement errors, or outliers can impact the validity of the results. When using large-scale longitudinal databases, assessments may occur at infrequent intervals. However, when possible, the use of regular, equally distanced assessments may improve reliability, enhance sensitivity to change, and allow for the identification of factors that account for that change [68]. Lastly, the interpretability of results should be considered, assessing whether the identified subtypes align with existing theoretical frameworks, have practical implications, or provide insights that advance the understanding in the field.

## 6. Conclusions

Overall, human aging is due to a combination of drivers from genetics to environmental factors, including individual’s lifestyle and exposure, leading to heterogeneous aging dynamics. This description drives the need to include the conceptualization of cognitive trajectories in the study of healthy aging. Different approaches to understanding cognitive trajectories have been proposed, either through modeling the aging population or understanding it at an individual level. In this paper, we highlighted the limitations associated with these methods. As an expansion, we proposed that subtyping trajectories is considered a viable alternative. In a normative approach, the average trajectory is calculated for the entire population. This approach aims to identify a generalized trajectory that represents the “typical” or “expected” cognitive aging process [69]. However, this approach can overlook the important variations and unique characteristics within the population by averaging out individual differences and considering only the averaged trajectory [70]. Differences that are deemed as noise or negligible within the normative framework may contain meaningful information about individual cognitive changes. Alternatively, an individualized approach focuses on analyzing each individual’s cognitive trajectory separately [25]. This approach includes the range of factors that can influence cognitive performance, such as sleep, caffeine intake, or nervousness [71], as well as contextual factors, such as distractions, location, and social company at the time of testing [72]. However, by focusing on individual measures, this approach may overestimate the weight of random fluctuations or temporary influences that do not reflect the underlying cognitive changes over time. The noise introduced by these factors can conceal the signal of true cognitive development or decline [68,73]. To address these limitations, a subtyping approach to cognitive trajectories in aging is proposed. This approach aims to create groups of individuals who have similar cognitive trajectories, effectively averaging out the negligible influences that may obscure the true signal (addressing the limitation of the individualized approach) [37]. By clustering individuals based on their trajectory patterns, we can identify distinct subtypes that capture the key differences in how people change in cognition over time (addressing the limitation of the normative approach). This approach recognizes population heterogeneity and acknowledges that cognitive aging is a complex process with individual variations while still providing a more fine-grained understanding compared to normative or individualizing approaches [7].

Subtyping cognitive trajectories relies on suitable early individual-specific detection methods that can lead to an improved understanding of changes in individual cognitive trajectories in healthy aging. Given the focus on the healthy aging population, it is crucial to study cognitive health in a longitudinal way to identify meaningful changes and determine appropriate intervention tools at actionable timescales. We believe that this approach will lead to a better understanding of the healthy aging process and the evolution of cognitive health at the individual level. This approach may be key in the identification of optimal opportunities for intervention in order to mitigate or reverse meaningful declines, increasing the health span of older adults [74,75,76]. Further, the inclusion of population subtyping can further lead to improved personalized recommendations and social prescribing programs. A limitation of the proposed work lies with the increased cost associated with the identification of subtypes as discussed in the work of [72]. These costs are tied with regular individual check-ups and an increased focus on methodological power. This increased initial cost could reflect a saving of cost in the treatment of later-life diseases, as these would be detected earlier and declines in cognition would be minimized or reversed.

Future work can benefit from understanding how subtypes within the healthy aging population are related to concepts such as brain and cognitive reserves [77,78], and how distinct trajectory subtypes are influenced by differences in reserve. Further, given the progressive nature of neuropathology, research that focuses on the conceptualization of subtyping trajectories within this context would also be beneficial to the scientific community. The proposed subtyping approach offers a promising alternative to normative and individualized models, with the potential to improve interventions and increase the health span of older adults. Here, we advocate that future work consider this approach in research and clinical practice and that doing so will lead to a deeper understanding of cognitive aging.

## Data Availability

No new data were created or analyzed in this study. Data-sharing is not applicable to this article.
